# Adherence to Immunosuppression Medications among Heart Transplant Recipients: Challenges, Opportunities, and Potential Role of Digital Approaches in the COVID-19 Era

**DOI:** 10.3390/jcdd8060068

**Published:** 2021-06-10

**Authors:** Tasmeen Hussain, Keira Nassetta, Sherif M. Badawy

**Affiliations:** 1 Division of Internal Medicine, Northwestern University Feinberg School of Medicine, Chicago, IL 60611, USA; tasmeen.hussain@northwestern.edu; 2 Department of Pediatrics, Northwestern University Feinberg School of Medicine, Chicago, IL 60611, USA; knassetta@luriechildrens.org; 3 Division of Hematology, Oncology and Stem Cell Transplantation, Ann & Robert H. Lurie Children’s Hospital of Chicago, Chicago, IL 60611, USA

**Keywords:** adherence, compliance, immunosuppression, heart transplant, digital, mHealth, eHealth, COVID, COVID-19, pandemic

## Abstract

Society and medical practice have been restructured dramatically to avoid further spread of the COVID-19 virus; telehealth/telemedicine, mask wearing, and nationwide social distancing practices have become widespread. However, we still face unprecedented challenges in fields where patients require frequent and active follow-up visits for monitoring, including that of solid-organ transplant, and in particular, heart transplant. Adherence to immunosuppression remains a unique challenge in heart transplantation, especially during the COVID-19 pandemic. Failure to adhere to immunosuppression can have disastrous consequences, including graft rejection and death. In this article, we discuss challenges related to adherence to immunosuppression medications among heart transplant recipients, as well as opportunities to leverage digital approaches and interventions to monitor and optimize adherence behavior and health outcomes in this population.

## 1. Introduction

As of April 2021, over 148 million people in the world have contracted COVID-19, and over 3 million have died as a result [1]. Society and medical practice have been restructured dramatically to avoid further spread of the virus; telehealth/telemedicine, mask wearing, and nationwide social distancing practices have become widespread. However, we still face unprecedented challenges in fields where patients require frequent and active follow-up visits for monitoring, including that of solid-organ transplant, and in particular, heart transplant recipients.

## 2. Effects of COVID-19 Pandemic on Transplants and Cardiovascular Health

Multiple aspects of organ transplantation have been affected by COVID-19. In the USA, a sample of 19 organ procurement organizations reported decreased rates of organ authorization by 11%, organ recovery by 17%, and number of organs transplanted by 18% for the period of March to May 2020, when compared to the same time frame in 2019 [2]. Centers are developing up to weekly COVID-19 testing regimens for patients on heart transplant waiting lists to optimize safety for patients and members of medical staff [3].

Despite these definite hurdles, obtaining an organ is just the first of many challenges. Post-transplant, the need for immunosuppression and frequent in-person follow-up puts these patients at increased risk of acute infection. A German study that followed 21 heart transplant recipients (HTxR) with COVID-19 found that eight patients developed severe disease requiring mechanical ventilation, and seven out of eight patients (87.5%) eventually died, which was associated with right ventricular (RV) dysfunction, arrythmias, and thromboembolic events [4]. Similarly, a study performed in northern Italy showed that compared to the general population, HTxR had a much higher prevalence of COVID-19 infection (18 vs. 7 cases per 1000) and related case fatality rate (29.7 vs. 15.4%) [5], highlighting the vulnerability of this population. Even healthy patients with COVID-19 have experienced a myriad of cardiovascular complications; one case series reports cardiogenic shock, ventricular arrythmias, and bradycardia and cardiac arrest requiring extracorporeal membrane oxygenation [6]. These complications could be just as catastrophic, if not worse, in a HTxR patient with a cardiac graft organ.

## 3. Challenges of Adherence to Immunosuppression Medications

Adherence to immunosuppression remains a unique challenge in heart transplantation, especially during the COVID-19 pandemic. Successful adherence is dependent on the patients’ ability to understand their regimens, obtain their medicines, and take them in the prescribed manner [7]. Failure to adhere to immunosuppression can have disastrous consequences, including graft rejection and death [7,8,9]. Immunosuppression may also be temporarily held as a recommendation from a medical provider, as is the case with many heart transplant patients who may have contracted COVID-19 or other serious infections [10].

Whereas the medical effects of COVID-19 are several, the social effects of the pandemic may lead to a number of additional challenges related to adherence behavior. Poor psychosocial functioning [11], lack of social support [12] and worse mental health [13] are known correlates to low adherence to immunosuppressants among adult heart transplant recipients. Additionally, immunosuppressants can be costly with the need for insurance coverage, and taking these medications as recommended requires a high level of health literacy to incorporate effectively in daily routine.

Further, the measurement of adequate levels of immunosuppression can be complex for the patient and provider. Traditionally, immunosuppressant drug level monitoring was the norm. One newer method is through the Cylex Immuknow assay, which utilizes a blood sample to measure cell-mediated global immunity, or T-helper lymphocyte response to mitogenic stimulation. This unique assay underwent moderate exploration, though initial data called into question its efficacy for suggesting infection and rejection risk [14].

Pediatric heart transplant recipients face additional barriers to immunosuppressants adherence. COVID-19 has disrupted the traditional school schedule with virtual home schooling being the expected model. Children, adolescents, and young adults spend the majority of their time performing school activities and homework online. As a result, there is a lack of in-person social interaction and support. Furthermore, pediatric patients already have difficulty adhering to strict medical regimens due to a natural desire for independence, and the desire to “fit in” without an “illness” label [9]. The need to frequently attend clinic visits and cooperate with caregivers may be seen or perceived by children and adolescents as annoyances, which may lead to strain amongst families and the healthcare team. Additionally, it may be more difficult to monitor and detect low or non-adherence in the pediatric population, as children and young adults are more swayed by social desirability, or the impulse to report higher adherence than is their true practice or adherence behavior [9].

On a positive note, the pandemic may have limited pediatric access to illicit substances and increased social connectedness with family members [15]. Furthermore, a 2017 systematic review of 19 articles outlining texting and mobile app interventions in pediatric patients showed that most studies showed feasibility and acceptability of interventions, and about half (8/19, 42%) showed significant improvements in preventive health behaviors. Strategies from such studies could be used to connect with pediatric transplant patients who require consistent care in the COVID-19 era [16].

## 4. Potential Role of Digital Approaches in Optimizing Adherence and Outcomes

**Figure 1:**
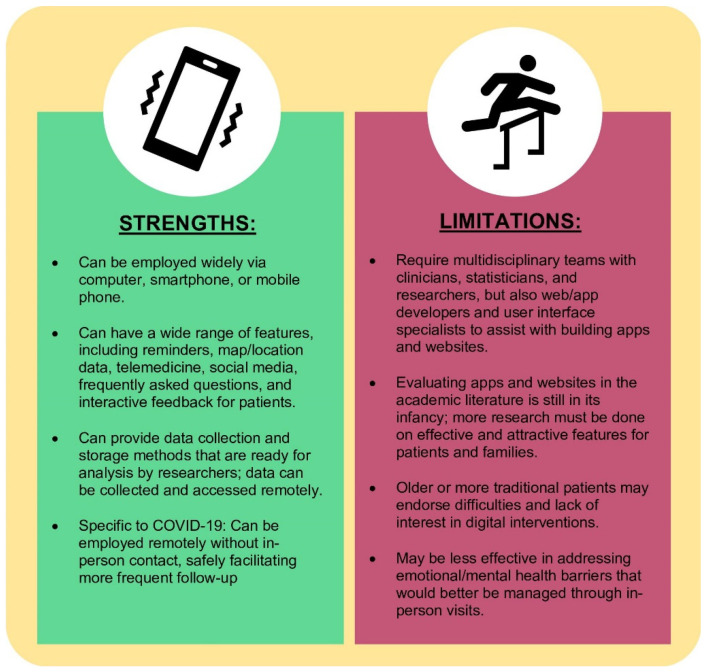
Strengths and limitations of using digital approaches in heart transplant.

With physical limitations imposed by the COVID-19 pandemic, personal technology represents a novel and effective approach to improve adherence among heart transplant recipients (Figure 1). Digital behavioral interventions, in particular mobile phone apps, have already been employed and implemented in a number of complex health conditions [16,17,18,19,20] including chronic disease in pregnancy [21], sickle cell disease [22,23], thalassemia [24], oncology [25], and heart disease [26]. In conjunction with text messaging and web-based interventions, healthcare providers can, in a more effective way, reach patients who would otherwise require frequent in-person appointments. Smartphones, tablets, and other avenues are ideal tools to leverage digital interventions as they are widely available [27] and can perform a broad variety of functions. One scoping review found that just in the first six months of the pandemic, between January to June 2020, 543 articles were published regarding telehealth, mostly in English (533/543, 98.2%) and in high-income countries (470/543, 86.6%), pointing to high levels of activity in this field of research [28].

In terms of aspects of care, digital interventions can fulfill multiple roles. First, mobile apps and text messages can provide timely reminders to take medications, prompt patients to pick up medications from designated pharmacies, and/or offer information on limiting costs. Many online platforms can give detailed information on medications and their side effects as well as lists of future appointments. Mobile and smartphones can also facilitate telemedicine visits with the added utility of transferring medical information to and from the healthcare team, and even from a third party if needed (e.g., laboratory or blood testing facility). Mobile apps can also be interactive by providing answers to frequently asked questions or giving feedback on symptoms and mental/emotional barriers to adherence. Finally, digital interventions can allow for communication among heart transplant recipient patients and/or their caregivers in support groups and community organizations, which is otherwise lacking in the era of COVID-19.

Researchers and clinicians or healthcare providers may also benefit from the use of novel personal technologies in the care of heart transplant patients. Websites, apps, and text messaging excel at handling data in a quick and reliable way. While telemedicine relies on clinician input to advise patients, interactive apps and websites can use pattern recognition or machine learning to “respond” to patient inputs and notify a care team member if concerning data is entered. Furthermore, servers that power the backends or databases of mobile apps and websites can offer data collection and storage capabilities to assist researchers addressing specific research questions. As heart transplant patients are a relatively small cohort, the wide dissemination of mobile apps and websites can help with data collection at multiple sites, allowing for more robust analyses and strong inferences of research findings with better generalizability.

There are several challenges inherent to the widespread implementation of digital technologies. For example, finding effective mobile (mHealth) and ubiquitous health (uHealth) interventions requires thorough and rigorous evaluation. While commercial apps and web-based interventions have been around for at least 20 years, academic literature regarding these approaches remains in its infancy and usually lags behind the pace of technology by 5–6 years on average. Furthermore, in-depth research in this area requires a multidisciplinary approach between clinicians, researchers, statisticians, app/website developers, and human factor experts in order to make a validated product. Institutions that work to establish formal, collaborative, and multidisciplinary teams will likely be the first ones to endorse and disseminate effective technologies to patients and families. Nevertheless, the cost-effectiveness of these interventions remains unclear [29,30]. We hope that technologies to improve adherence and medical knowledge can then translate to durable, long-term improvements in quality of life, health outcomes, and survival among pediatric and adult heart transplant recipients.

## 5. Innovation in Digital Approaches

The COVID-19 pandemic arrived at a truly unique time in academic medicine, when innovations in technology are already undergoing deserved exploration. Multiple unique aspects of care can be incorporated into mobile apps and other personal technologies. For example, adherence measurement can be hugely augmented by technology. Pill bottle monitors have emerged alongside reminder mobile apps to help patients schedule and take medications as part of complex regimens. For patients who require assistance with travel or are too sick or immunocompromised to safely pick up medicines, a voucher or delivery system would be enormously helpful. Finally, effective online appointment software and additional telehealth or telemedicine options could assist providers in giving timely feedback on vital signs and examination data gathered at home, allowing more patients to receive appropriate follow-up. These approaches are all novel and necessary in the setting of the COVID-19 pandemic.

Multiple approaches to promote adherence behavior in solid-organ transplant regimens have been published in the last year [31,32] which may translate to the heart transplant literature. A meta-analysis by Shi et al. in October 2020 reviewed 27 articles regarding adherence-enhancing interventions in solid organ transplant recipients. The authors reported a promising correlation between the use of interventions and higher patients’ self-reported adherence, but interestingly not with immunosuppressant drug levels, suggesting further research is necessary [33].

In the field of heart transplants, research is just beginning to flourish. Gomis-Pastor et al. published one of the first studies on an mHealth intervention to improve immunosuppression adherence in heart transplant patients [34]. The utilized intervention, called “mHeart”, was a mobile app connected to the hospital information system with a variety of functions, including answering patients’ questions, empowering patients in terms of self-care, and sharing professionals’ recommendations. Using mHeart was associated with a significantly improved adherence to immunosuppression, with an increase in adherence rate from 61 to 87%, as measured by the validated Simplified Medication Adherence Questionnaire (SMAQ) [34]. Additionally, a protocol published by Lieb and colleagues in October 2020 for German heart transplant patients outlined a prospective observational trial to assess mental and emotional barriers to medication adherence, as well as potential correlated lifestyle behaviors. Non-adherence is to be measured through an electronic pillbox as well as phone-based self-reports, which both will be performed remotely. Given the diverse methodology of these articles, further research in this area continues to be necessary [35].

## 6. Conclusions

The COVID-19 pandemic remains an unprecedented set of circumstances, especially for our heart transplant patients. Clinicians and other healthcare providers should remain vigilant to identify both social and medical impacts of the pandemic, especially in relation to medication adherence and health outcomes. We believe that necessity drives the creation and evaluation of technology-based systems and various digital approaches moving forward, with the goal of improving care now during the pandemic era as well as in the future.
